# Exploring interrelationships of COVID-19 dimensions in Saudi Arabia: a systematic review

**DOI:** 10.1186/s42269-023-01041-w

**Published:** 2023-05-26

**Authors:** Hamad Mansur Aldossari

**Affiliations:** Geography and Geographical Information Systems Department, Imam Mohammad Ibn Saud Islamic University (IMSIU), Riyadh, Saudi Arabia

**Keywords:** Vaccine hesitancy, Psychological impact, Nutrition, Awareness, e-Learning

## Abstract

**Background:**

COVID-19 affected the world threatening medical, social, economic and community dimensions. Along with the other countries of the world, Saudi Arabia also witnessed critical emergencies leading to serious disaster due mainly to the overcrowding at certain urban residential localities. Nevertheless, the situation handled meticulously not only with measures and combating strategies but also with documentations and researches to build sustainable confrontation systems and lateral programs.

**Main body of abstract:**

This attempt is a consolidation of the academic efforts on COVID-19 in the country aiming to contribute to the knowledge base aiding to future planning on preparedness. An online survey of published academic research from peer reviewed journals were carried out during August-December 2022, using COVID-19 in Saudi Arabia as search criteria. Many areas of concern are assessed in the context which are classified into spread and burden, patient statistics, symptoms and other clinical dimensions, vaccinations and vaccine acceptance/hesitance, psychosocial impact, impact on education, impact on health staff, impact on migration, impact on nutrition, and control measures adopted.

**Short conclusions:**

A consolidation of these research findings explains the scientific academic community alertness in raising up to the emergency pandemic situation, which facilitated strategy and policy formulations by the Government ministries and other governing bodies. These researches are linked to stagewise programmatic efforts to curtail the epidemic as a mode of accomplishments.

## Background

### Main text

The epidemic of COVID-19 gave a diverted epidemiological research, worldwide, to focus on emergence of new diseases of infection intensity beyond the traditional data trend and pattern analyses and determinants to reveal interconnections. Most of these analyses were carried out at national levels, although there are few international comparisons. One of the countries that witnessed serious spread, in the early days which controlled to the maximum with futuristic sustainable programs is Saudi Arabia. Both the programmatic health measures and the community level programs aided to create awareness paving way for support systems started with the Riyadh declaration on digital health during August, 2020 as a landmark innovation for resilient global emergency public health care systems (Al-Knawy et al. 2020; Al-Otaibi 2020).

A massive infodermic (an overabundance of information), by various professionals, partially accurate, turned social media to spread myths and rumors (Barry et al. 2020). Moreover, there were efforts exploring medical, clinical, and socioeconomic dimensions at national, local and grassroots levels reflecting developments, movements, activism, and humanitarian supports, all over the country to save people from disasters, depressions, disappointments, and despair. An effort is made to consolidate those researches to disseminate for a better understanding and interlinking with strategic approaches. It is hoped that such a consolidation serves the knowledge society in precautions, preparations, policies, and intervention strategies.

## Data and methods

A review of published researches, specifically on Saudi Arabia available online, since 2020 were reviewed, summarized, and consolidated to view the direction and spread of COVID-19 research in the country. The core contents and data of these researches were extracted through online search during August–December 2022 selecting COVID-19 in Saudi Arabia as the search criterion. All available research studies published on academic journals were selected, reviewed and included relevantly. Those out of the context such as explanations, case studies, laboratory experimentations and clinical trials were excluded.

### Major issues addressed

This review brought to notice many published researches: all of them are classified and categorized for clarity of discussion and consolidation to aid strategic interventions.

1. Spread and burden

COVID-19 spread in the country through borders of eastern region, during March 2020, enroot Iran and China caused infection from one administrative area to others, especially the highly populated geographic locations such as Riyadh, Makkah and Madina (Salam et al. 2022a, 2022b). Thereafter, it spread all over the country rapidly, creating morbidity, mortality, and health care burden. But with greater strategic efforts, the disease spread was controlled to a large extent. One of the first strategies being implemented is the Riyadh declaration on digital health launched in mid 2020 paved way for smooth transfer of access to health care from personal to virtual platform institutionalizing data-based actionable health systems placing individual and population health at the forefront of future endeavors (Al-Knawy et al. 2020). This has telemental health provision and virtual outpatient clinics with well defined service scope and impact horizons that equip trainees with skills and quality to deliver services including e-prescriptions (Banjar and Alfaleh 2021; Alabdulkarim et al. 2020). Protective measures, awareness campaigns, transmission routes, personal hygiene are measures to combat this epidemic (Bazaid et al. 2020).

2. Patient statistics

There are many statistics on spread of infection including case fatality in Saudi Arabia (Alabdulkarim et al. 2020); demonstrating old age (above 60 years) is a high-risk group (Asdaq et al. 2022). Studies examined symptoms, root causes of infection, and major affected at-risk groups including international passengers revealing non-adherence to precautionary measures such as mask, physical distance, and contact with people in isolation as reasons for infection (Jdaitawi 2020; Elawad et al. 2021). Socio-demographics of COVID-19 patients such as sex, age group, nationality, source of infection, employment, type of infection, and treatment dimensions were explored by various researchers (Jdaitawi et al. 2020; Elawad et al. 2021; Almarashi et al. 2021). Females, natives, early adults, and employed persons in direct contact are more affected. Treatment statistics show that most of them sought treatment, followed isolation advices, and adopted precautions.

3. Symptoms and other clinical dimensions

COVID-19, a variant of SARS (Severe Acute Respiratory Syndrome), virus infect to humans by direct contact with animals and later through human to human transfer, have serious impact on genetic diversity, mutation, and recombination (Eifan and Hanif 2020). It was stated by Alhujaili et al. (2021) that those who follow COVID-19-related news on a daily basis, those with no prior infection, and those having reported a prior psychiatric diagnosis are more susceptible to infections: a result implying and enabling healthcare professionals to be recognized as at high risk. Those who exhibit severe conditions, in Saudi Arabia, were foreigners who had a history of chronic diseases and co-morbidities including diabetes, hypertension, and thyroid (Alqahtani et al. 2020). Moreover, COVID-19 as an epidemic interferes with preventive, chronic and acute care visits (Shatla et al. 2021). It was recognized that those with diabetes, a significant health issue in Saudi Arabia, affected by COVID-19 are at high risk of disease severity and mortality (Robert and Al-Dawish 2021). Thus, COVD-19, the worst pandemic of the millennium, impact on healthcare system, especially of comorbid chronic conditions and cancer (Ibrahim et al. 2020; Khoshaim et al. 2020).

A clinical study of blood groups showed significant effects of old age and diabetes on the one hand and youths and men on the other as severely infected with COVID-19, which could be attributed to mobility, smoking and obesity (Badedi et al. 2021). The former group (old aged diabetic patients) during the pandemic period, faced many challenges of medical treatments and life style modifications as part of care strategies (Alshareef et al. 2020). Medication adventures, abnormal lipid profiles in the COVID-19 patients during clinical manifestations, effectiveness of hydroxychloroquine in symptomatology reduction, and even chemoprophylaxis are worth noting (Mohammedsaeed et al. 2020; Almazrou et al. 2021; Mirghani et al. 2021; Al-Tawfiq and Memish 2020). However, COVID-19 patients with cardiac diseases comorbidity, especially of old aged, seeking intensive care or critical care treatments often report adverse outcomes especially in compensation with tobacco smoking habit (Khan et al. 2020; Abohamr et al. 2020). Thus, essential healthcare services are critical during pandemic especially for elderly with hypertension, diabetes, respiratory diseases, cardiac diseases, and malignant neoplasms equipped to address their most reported symptoms, such as cough, fever, fatigue, different sputum production, and comorbidities of hypertension and diabetes (Al-Omaria et al. 2020; Alsofayan et al. 2022).

4. Vaccinations and vaccine acceptance/hesitance

With the launch of vaccine, there were varied responses from sectors, both in favor and against: acceptance being high from certain socio-demographic categories (determinants)—males, non-natives, and governmental sector employees have comparatively higher acceptance than others (Al-Mohaithef and Padhi 2020). However, vaccine side effect assessment revealed short-term effects including fatigue, pain at the vaccine site, fever, chills, and head ache, more so during the second doze: majority consumed medicine but with minimum physician consultations and hospitalizations (Alhazmi et al. 2021). Moreover, vaccine hesitancy is debated, especially among adults, due to confusions of efficacy and safety, thereby recommending vaccine literacy programs to overcome misconceptions (Almaghaslah et al. 2021). Although the Ministry of Health encouraged vaccinations against COVID-19 in order to maintain community immunity; refusals were high even after clarifying safety, reliability, and effectiveness (Alrajeh et al. 2021). Nevertheless, willingness to accept vaccine was higher among old aged, married persons, foreigners and those with high levels of education (Al-Mohaithef and Padhi 2020) whereas vaccine hesitancy has predicted demographics (age, gender and nationality), clinical trial sufficiency and identification of side effects (Zahid and Alsayb 2021).

A large majority of people are willing to accept vaccine as they have confidence and trust in government decisions and healthcare system perceiving the risk of COVID-19, which depends largely on interventions of the health authorities, medical practitioners, and other entities (Alqahtani 2022). Even, vaccine resistance was observed among healthcare workers (Qattan et al. 2021). Although, no major side effects or breakthrough infection of vaccine was reported, there were differences between males and females across age groups in reported symptoms (Al-Bahrani et al. 2021). Perceived susceptibility and benefit are important constructs in the intension to vaccinate which is associated with socio demographics including sex, nationality, education, work in healthcare, and monthly income apart from beliefs, campaign implementation, knowledge levels, and attitudes, (Habib et al. 2022; Alobaidi 2021; Al-Naam et al. 2022).

5. Psychosocial impact

New methods of learning and training lead to stresses among health staff and patients creating anxiety and depression, thus demanding psychological support programs (Balhareth et al. 2020). Additionally, an increase in mental health visits were reported during the pandemic (Shatla et al. 2021). Higher levels of depression and anxiety prevalence were reported among the general population of Riyadh city during the pandemic, especially among females, younger individuals and those with a history of psychiatric illnesses, that too, at a vulnerable level needing more support (Al-Hanawi et al. 2020). Such psychological distresses affected health workers engaged in COVID-19 duties too: higher among males but without affecting their daily life as they were merely of psychological distress caused by long working hours (Alqutub et al. 2021). COVID-19 pandemic negatively affected individual quality of life and psychological health (Algahtani et al. 2021). Thus, psychological interventions for general and vulnerable population implemented in combination with pandemic response at an early stage, as recommended by Alkhamees et al. (2020), add value.

In comparison, foreign nurses have greater awareness, positive attitudes, optimal prevention, and positive perceptions and thus the native staff need empowerment through prioritized training programs to handle cases precisely (Al-Dossary et al. 2020). This pandemic affected the home atmosphere due to online education, work, and entertainments, which were formerly of out of home activities causing tensions of privacy and hospitality (Al-Khateeb and Peterson 2021). Health workers, youths, females, and private sector employees are at distress due to COVID-19, thus, urging psychological supportive programs to mitigate (Al-Hanawi et al. 2020). Such impacts are evident in health care staff, even on physicians (Al-Sulais et al. 2020).

6. Impact on education

Educational days lost has been compensated with e-learning methods, which faced serious constraints due to its initiation (Hoq 2020). This educational method has faced with both acceptance and rejections, thereby developing smart learning methods leading to delays in examinations, experimenting alternative methods such as webinars, recorded sessions, and social media but mostly depending upon the adaptability of students, parents and educators (Balhareth et al. 2020; Khoshaim et al. 2020). These platforms are expanded to improve awareness through procedures adhering to precautionary measures (Zitoun 2020). It is urged to the educational institutions to improve their online learning platforms to make them more student friendly (Madhesh 2021).

A study of dental interns reveals the impact of virtual leaning on preparedness towards clinical rotations and decreasing confidence affecting their study and work at hospital apart from patient management, cost of precautions, and psychological burden (Mohsin et al. 2022;. Al-Qahtani et al. 2022). Pharmacy education also had suffered in the period due to the dependence on online services (Almetwazi et al. 2020). Services necessary for awareness programs, health education advices, online counselling were also contributed by dentists (Attar 2021). Internships of all disciplines are impacted (Bugis 2020).

An analysis explained negative and positive academic perceptions highlighting balancing of workload and improving technical support to reduce perceptions affecting satisfaction during the pandemic (Hassan et al. 2021). It is urged to keep the role of Saudi Arabia in the Islamic world that the efforts to address the pandemic beyond political, monetary and social concerns to ensure safety of population (ElSayed 2020). Children, school going, are severely affected by the pandemic due to disruptions in schooling. Although, emotional maltreatment reduced, neglect increased (Alenezi et al. 2022).

7. Impact on health staff

Increased adverse psychological impact revealed among healthcare workers during COVID-19 include anxieties and stress due to risk of infection, high workload, stigma, and lack of necessary equipment (Alamri et al. 2021). They are also influenced by the stress, depression, and anxiety of wellbeing depending upon income level, tolerance, and trust (Alenzi et al. 2022; Alkhamees et al. 2020). Anxiety and depression symptoms exhibited by healthcare staff vary in severity, which explains the importance of mental health in combination with physical needs including sleep (Al-Ateeq et al. 2020). Healthcare workers of Saudi Arabia, on the other hand, are well equipped to handle epidemic with their knowledge and experience gained through prior campaigns conducted to handle MERS-CoV in protective hygiene and anxiety reduction (Temsah et al. 2020).

8. Impact on migration

One of the researches on migrant health during COVID-19 explains the challenges of healthcare system facilitating residents including vulnerable migrants at high risk of acquiring and spreading COVID-19 infection (Ali et al. 2021). Besides, immigrants are founded to be more prone to infection due mainly to the crowded living arrangements, and so culminating overcrowded living spaces is necessary to reduce infection rate (Alqahtani et al. 2020). Other measures adopted to foreign labor force include imposition of strict guidelines of housing accommodation and repatriation programs in accordance with information for tackling the disease or other diseases, elsewhere (Nurunnabi 2021).

9. Impact on nutrition

There are many studies on nutrition intake during COVID-19. One of them giving hope for future is a positive nutrition practice explaining a change in dietary patterns of Riyadh residents: home cooked meals with quality and quantity as per healthy food choices and nutritional balance (Alhusseini and Alqahtani 2020).

10. Control measures adopted

Virtual clinic initiatives, established at outpatient clinics, facilitated e-prescriptions (Alabdulkarim et al. 2020). Cancellation of UMRA and HAJJ (Ebrahim and Memish 2020a, 2020b; Shatla et al. 2021) was also of importance. Moreover, it is realized to maximize knowledge to formulate public health policies for controlling epidemic (Jdaitawi et al. 2020). One of the important strategies of health care administration giving hope is the decree ensuring all residents (including visa violators) receive free COVID-19 treatment without any legal or monetary repercussions, through telephone calling to the Ministry of Health established good physical and mental hygiene and shift to a new normal status of wellbeing (Al-Humaid et al. 2020). Moreover, effective communication, early intervention, and alternative learning methods are implemented to prevent further harm in the crisis period (Alyami et al. 2020). In addition, Saudi Arabia played a key role, internationally, on humanitarian grounds to support communities affected by COVID-19 by way of exchanging shipments of medical equipment, medicines, and other aides (Meo 2020). Recommendations are made by the Ministry of Health, to execute strategies to reduce comorbidity prevalence through studying actual burden with different demographic characteristics (Asdaq et al. 2022).

Strategic actions, step by step, implemented by Ministry of Health, in Saudi Arabia enabled control of the epidemic (Yezli and Khan 2020). Community health and wellness programs focusing on fulfilling individual needs to live healthy and combating psychological effects of the crisis were important and to be implemented to deal with this local and global impact of COVID-19 (Algahtani et al. 2021). Moreover, the tele-counseling network established by the Ministry of Health proved to be highly efficient as a supplementary voluntary care and counseling strategy (Alhraiwil et al. 2022). The wellbeing framework is placed at great risk forcing public authorities to enforce isolation and other extreme measures, where the adverse effects are many including pressures, dread, disarray etc. (Alsharif 2021).

Pharmaceutical sector also went through amendments to offer medicines at doorstep and online counselling as part of the reorganization of global health care (Ahmad et al. 2020). Their underutilization have negative impact, and so enhancing the role of and contribution of pharmacists in patient care management at hospitals under healthcare crisis conditions are of importance (Assiri et al. 2020). With all such limitations and restrictions to contain and control the epidemic, Saudi Arabia confidently moved to open up the borders, trade, travelers, and pilgrims (Al-Ahdal 2020).

**Fig. 1:**
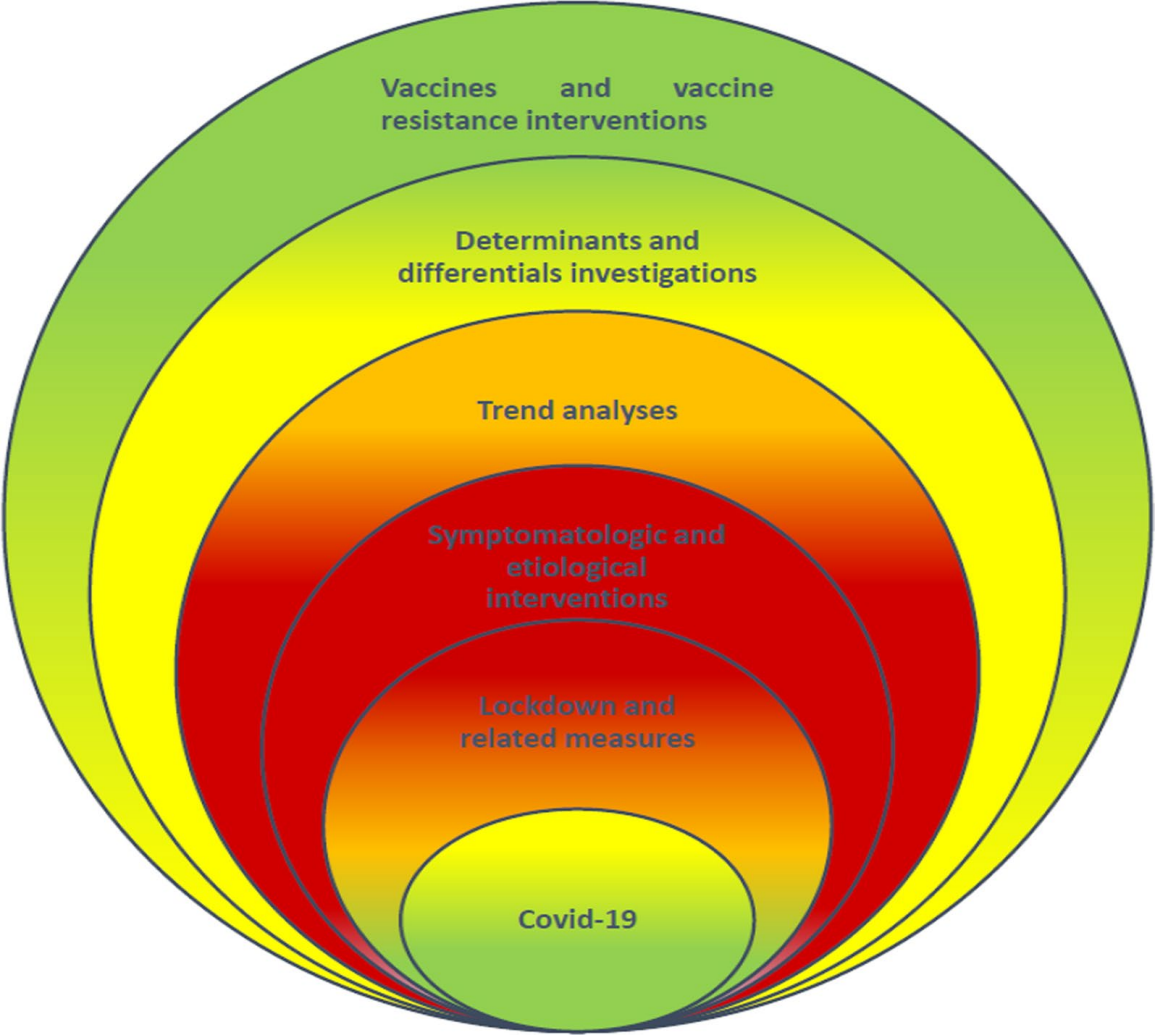
COVID-19 dimensions and stage wise accomplishments

In short, Saudi Arabia’s fight against COVID-19 can be traced through literature starting from the first infection at Eastern Region location to its spread all over the country causing burden of human lives of around 10.000 and morbidity of around one million cases. COVID-19 in Saudi Arabia has been through several phases as shown in Fig. 1. Starting on March 2020, at the beginning of infection in the country having a high infection strength, it continued the brutal spread. During this phase, there were several strategies to control infection in line with lockdowns, restrictions and cancellations, as researches of that period reveals. This phase was a green phase with very low level of infection. Slowly this stage moved to a yellow phase, within a month or two, where infection rate increased turning the green to yellow (a phase of caution). Along with change in the epidemic situation, there were changes in academic investigations to symptoms and causes in order to implement intervention strategies. This phase turned to be orange (a phase of seriousness) and slowly red (a phase of danger). Researchers started to analyses the data for trends in order to estimate and predict future. While this phase was hopeful for a lesser spread, there were waves of second and third, intermittently, challenging the healthcare delivery system. Researchers turned to be more advanced looking at the determinants and differentials. With this phase a control obtained and the vaccine started launching. Researches started moving to another stage of vaccine administration, impacts, prejudices, efficiency, side effects, and future complications. This phase has taken a long time but has converted red to orange and thereafter to yellow and finally to a green stage characterized by life style re-gained. Overall, this review of literature explains the whole dynamics of COVID-19 in Saudi Arabia.

## Conclusions

Many researches were carried out in Saudi Arabia in the context of COVID-19 to explain dimensions of significance. Majority of the efforts focus of medical, clinical and psychosocial aspects. There are continuous efforts and scientific processes in the country’s academia, which shall boost the disaster preparedness and epidemic control and awareness building in such emergency situations. No doubt, researchers and academic attempts are encouraged through funding and infrastructure networking.

## Data Availability

All the data referred and discussed in the manuscript are cited in the text with its references listed.
